# Mechanisms and Functions of Sweet Reception in Oral and Extraoral Organs

**DOI:** 10.3390/ijms25137398

**Published:** 2024-07-05

**Authors:** Ryusuke Yoshida, Yuzo Ninomiya

**Affiliations:** 1 Department of Oral Physiology, Graduate School of Medicine, Dentistry and Pharmaceutical Sciences, Okayama University, Okayama 700-8525, Japan; yoshida.ryusuke@okayama-u.ac.jp; 2 Faculty of Medicine, Dentistry and Pharmaceutical Sciences, Okayama University, Okayama 700-8558, Japan; 3 Graduate School of Dental Science, Kyushu University, Fukuoka 812-8582, Japan; 4 Monell Chemical Senses Center, Philadelphia, PA 19104, USA

**Keywords:** sweet taste, energy homeostasis, T1R3, GLUT, SGLT, sugar

## Abstract

The oral detection of sugars relies on two types of receptor systems. The first is the G-protein-coupled receptor TAS1R2/TAS1R3. When activated, this receptor triggers a downstream signaling cascade involving gustducin, phospholipase Cβ2 (PLCβ2), and transient receptor potential channel M5 (TRPM5). The second type of receptor is the glucose transporter. When glucose enters the cell via this transporter, it is metabolized to produce ATP. This ATP inhibits the opening of K_ATP_ channels, leading to cell depolarization. Beside these receptor systems, sweet-sensitive taste cells have mechanisms to regulate their sensitivity to sweet substances based on internal and external states of the body. Sweet taste receptors are not limited to the oral cavity; they are also present in extraoral organs such as the gastrointestinal tract, pancreas, and brain. These extraoral sweet receptors are involved in various functions, including glucose absorption, insulin release, sugar preference, and food intake, contributing to the maintenance of energy homeostasis. Additionally, sweet receptors may have unique roles in certain organs like the trachea and bone. This review summarizes past and recent studies on sweet receptor systems, exploring the molecular mechanisms and physiological functions of sweet (sugar) detection in both oral and extraoral organs.

## 1. Introduction

Taste plays a critical role in food intake by creating sensations that are either preferable or unpreferable. Innately, sweet, salty (at low concentrations), and umami tastes evoke preferable sensations, whereas sour, salty (at high concentrations), and bitter tastes produce unpleasant sensations. Thus, the taste system in the oral cavity functions as a gatekeeper, determining whether the food in the mouth should be ingested or not. Sweet taste, induced by sugars, signals the presence of a carbohydrate source of calories, thereby strongly linking it with energy metabolism. There are two types of receptor systems for sweet taste on the tongue. One is the sweet receptor TAS1R2 + TAS1R3 (taste receptor family 1 members 2 and 3), which is broadly responsive to sugars, artificial sweeteners, and even sweet proteins. The other is the glucose transporter, which specifically detects sugars. The molecular mechanisms of sweet reception in sweet-sensitive taste cells are now generally understood. However, the sensitivity of these cells is not constant; it is regulated by external and internal factors such as temperature and hormones.

Recently, numerous studies have focused on the functions of extraoral taste receptors. Sweet taste receptors, including TAS1Rs and glucose transporters, are expressed in various organs such as the gastrointestinal tract, pancreas, and brain. These receptors play roles in glucose absorption, insulin release, sugar preference, and food intake, all of which are crucial for maintaining energy homeostasis in the body. Thus, sweet receptors act as energy sensors within the body. Additionally, sweet receptors may have unique functions in certain organs, such as defending against bacterial proliferation in the trachea and contributing to bone remodeling in bones.

In this review article, we summarize past and recent studies on sweet receptor systems and discuss the molecular mechanisms and physiological functions of sweet detection in both oral and extraoral organs.

## 2. Mechanisms for Sweet Reception

**Figure 1:**
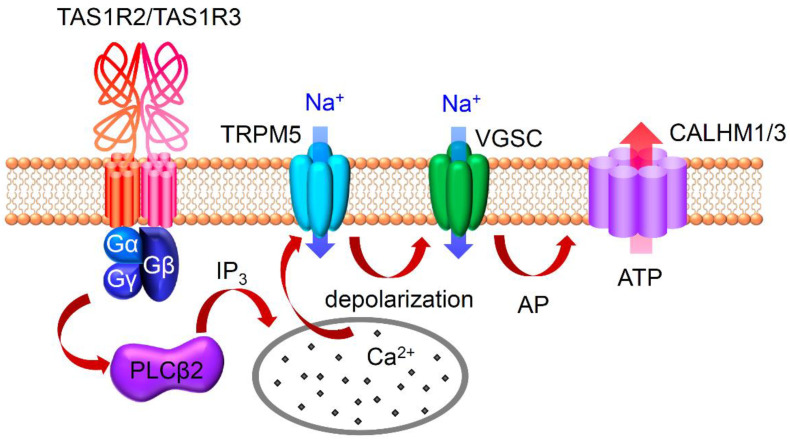
Schematic diagram showing molecular mechanisms for sweet detection via TAS1Rs in taste cells. The binding of sweeteners to TAS1R2/TAS1R3 activates a trimeric G-protein (Gα-gustducin, Gβ1 or Gβ3, and Gγ13) and phospholipase Cβ2 (PLCβ2). Then, inositol-1,4,5-trisphosphate (IP_3_) is produced, and [Ca^2+^]_i_ is increased by Ca^2+^ release from Ca^2+^ store. Ca^2+^ activates the transient receptor potential channel M5 (TRPM5), leading to cell depolarization and the firing of action potentials (APs) via voltage-gated sodium channels (VGSCs). Then, ATP-permeable CALHM1/3 opens to secrete ATP from the taste cell.

Prior to the discovery of the sweet taste receptor TAS1R2/TAS1R3, genetic studies demonstrated that the *Sac* and *dpa* loci on mouse chromosome 4 influenced the response of taste nerves and behaviors towards sweet tastants. This indicated an association between these loci and sweet taste receptors. The *dpa* locus was determined by investigating mouse strain differences in gustatory nerve responses and behavioral responses to a sweet-tasting amino acid, D-phenylalanine [1,2,3,4,5]. The *Sac* locus was implicated in affecting behavioral preference among saccharin and some other sweeteners [6,7,8,9]. Subsequently, genes encoding components of sweet receptors (*Tas1r2* and *Tas1r3*) and also an umami receptor component gene (*Tas1r1*) were identified on these loci of mouse chromosome 4 [10,11,12,13,14,15,16]. The deletion of the *Tas1r2* and/or *Tas1r3* gene in mice resulted in diminished gustatory nerve and behavioral responses to sweet compounds, emphasizing the crucial role of both TAS1R2 and TAS1R3 in sweet tastant detection [17,18]. TAS1R2 and TAS1R3 belong to the class C G-protein-coupled receptor family, featuring a large extracellular amino-terminal domain (ATD) known as the Venus flytrap module (VFTM), a cysteine-rich linker domain (CRD), and a transmembrane domain (TMD). The primary binding site for sweet compounds resides in the VFTM. Glucose and sucrose were shown to bind to the VFTM of both TAS1R2 and TAS1R3 through binding assays using a purified ATD of mouse TAS1R2 and TAS1R3 [19,20]. Species differences in sensitivity to sweet compounds led to the identification of the binding site for artificial sweeteners such as aspartame and neotame within the VFTM of human TAS1R2 [21,22,23,24]. This site is also crucial for binding with D-tryptophan, saccharin, acesulfame K, and sucralose [25]. Additionally, the TMD of human TAS1R3 is essential for binding to cyclamate [21,26,27]. Moreover, various sweet proteins such as Brazzein, Mabinlin, Miraculin, Monelin, Neoclin (Curculin), and Thaumatin could bind to TAS1R2/TAS1R3 [28,29,30,31,32,33,34,35], highlighting the receptors’ ability to interact with a wide range of sweet compounds. The binding of a sweet tastant to TAS1R2/TAS1R3 triggers conformational changes in this receptor, leading to the activation of a trimeric G-protein composed of Gα-gustducin (Gnat3), Gβ1 or Gβ3, and Gγ13 [36,37]. This activation subsequently triggers the activation of phospholipase Cβ2 (PLCβ2) to produce inositol-1,4,5-triophosphate (IP_3_), which binds to inositol-1,4,5-triophosphate receptor type 3 (IP_3_R3), resulting in the release of Ca^2+^ from the calcium stores [38,39]. The released Ca^2+^ activates transient receptor potential channel M5 (TRPM5), leading to cell depolarization and the firing of action potentials [40,41]. The mechanisms for sweet detection via TAS1R2/TAS1R3 are shown in Figure 1.

**Figure 2:**
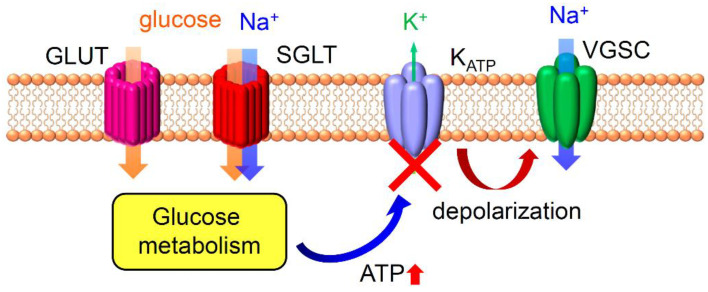
Schematic diagram showing molecular mechanisms for sweet detection via glucose transporters in taste cells. Glucose entering via glucose transporters (GLUTs) and/or sodium–glucose transporters (SGLTs) is metabolized to produce ATP. The activity of K_ATP_ channels is inhibited (indicated by X) by an increase in [ATP]_i_ (indicated by red up arrow), leading to cell depolarization. Na^+^ entry through SGLTs also induces cell depolarization. Such depolarization activates voltage-gated channels such as voltage-gated sodium channels (VGSCs).

On the other hand, numerous studies have proposed the existence of a sweet detection system, particularly for sugars, distinct from TAS1R2/TAS1R3. For example, mice lacking *Tas1r3*, *Gnat3*, or *Trpm5* exhibit reduced responses to various sweeteners, yet they still display residual responses to several sugars, notably glucose [17,36,42]. Gurmarin, isolated from the *Gymnema sylvestre* plant, selectively inhibits mouse TAS1R2/TAS1R3 [43]. Consequently, tongue treatment with gurmarin diminishes responses to sweeteners in mice. Nonetheless, mice retain gurmarin-insensitive gustatory nerve fibers that respond to sweet compounds [44,45,46]. An alternative mechanism for sweet reception, independent of TAS1R2/TAS1R3, could involve glucose transporters. Reports indicated the expression of certain glucose transporters (GLUTs) and a sodium–glucose cotransporter (SGLT1) in rodent taste cells [47,48,49]. Moreover, taste cells express subunits of the metabolic sensor K_ATP_ channel, including sulfonylurea receptor 1 (SUR1) and inwardly rectifying potassium channel 6.1 (Kir6.1) [47,50]. Based on these molecular expressions, a plausible sugar sensing mechanism can be proposed: (1) Oral glucose is transported into a taste cell via the glucose transporter. (2) Intracellular glucose undergoes metabolism, generating ATP within the taste cell. (3) ATP binds to the K_ATP_ channel, leading to channel inhibition (closure). (4) The closure of K_ATP_ channels, which are potassium channels, results in cell depolarization and the activation of voltage-gated channels. The mechanisms for sweet detection via glucose transporters are shown in Figure 2. However, this mechanism might be specific to monosaccharides since glucose transporters primarily transport monosaccharides, not di- or polysaccharides. Various α-glycosidases convert disaccharides into monosaccharides, and these enzymes, such as maltase-glucoamylase and sucrase-isomaltase, are selectively expressed in sweet taste cells [51]. This suggests that di- and polysaccharides can also activate taste cells through the digestion by α-glycosidases and the subsequent uptake of monosaccharides via glucose transporters. Actually, treatment with disaccharidase inhibitors such as miglitol and voglibose specifically reduced gustatory nerve responses to disaccharides [51]. Additionally, the involvement of glutamate transporters in sugar detection on the tongue was demonstrated through gustatory nerve recordings and cellular experiments. The treatment of the tongue with an inhibitor of sodium–glucose transporters, phlorizin, abolished the enhancement in sugar responses by NaCl, and the treatment of phlorizin or a glucose transporter inhibitor, phloretin, reduced the apical uptake of the fluorescent glucose analog 2-[N-(7-Nitrobenz-2-oxa-1,3-diazol-4-yl)amino]-2-deoxy-D-glucose (2-NBDG) into taste bud cells [52]. Thus, glucose transporter-dependent sugar detection in taste bud cells may play a role in sweet taste reception in the oral cavity.

## 3. Mechanisms for Sweet Modulation

Physicochemical factors exert a notable influence on taste sensitivity, with temperature being a significant contributor to the perception of sweetness. The perceived sweetness of sugar solutions is weak at cool temperatures and increases strongly with temperature [53,54]. This temperature dependency of sweet taste can be attributed to the properties of TRPM5, a crucial component in the transduction process for sweet taste. The activation of TRPM5 channels reached its peak at around 35 °C, and alterations in the responses of the chorda tympani nerve to sweeteners due to temperature changes were absent in mice lacking *Trpm5* [55]. Moreover, recent findings suggest that voltage-gated sodium and potassium channels in type II taste bud cells (sweet, bitter, and umami cells) also have thermal sensitivities, which affect the generation of action potentials [56]. Thus, temperature plays a pivotal role in modulating the responsiveness of sweet-sensitive taste cells through its effects on ionic conductance mechanisms.

Taste sensitivity is also regulated by humoral factors depending on the internal state of our body. Some hormones and bioactive substances are known to affect sweet taste sensitivity by acting on peripheral taste receptor cells. The most well-studied factor is leptin, an anorexigenic hormone produced by adipocytes, which plays a significant role in regulating food intake, energy expenditure, and body weight [57]. Mutant mice with defects in the *ob* gene (*Lep*, encoding leptin) or the *db* gene (*Lepr*, encoding leptin receptor) displayed severe obesity, increased appetite, and diabetes [58,59]. Studies on gustatory nerve responses in *db*/*db* mice, which lack functional leptin receptors, revealed enhanced responses to sweet compounds compared to wild-type (WT) mice, while responses to salty, sour, and bitter compounds remained largely unchanged [60,61,62]. This increased sensitivity to sweetness in *db*/*db* mice was not solely due to their diabetic condition, as mice induced to be diabetic by the administration of streptozotocin did not show such increased responses to sweet compounds [60]. In addition, preference tests further support the heightened attraction to sweeteners in *db*/*db* mice compared to lean controls [60]. Thus, the *db* gene, responsible for the leptin receptor deficiency, appears to play a role in sweet taste sensitivity in mice. The administration of leptin to lean WT mice reduced gustatory nerve responses to sweet compounds but had no effect on responses to other tastes [63], highlighting the role of leptin in modulating sweet taste sensitivity. Of course, this suppressive effect of leptin was not observed in *db*/*db* mice due to their lack of functional leptin receptors. Consistent with nerve response findings, behavioral assays also demonstrated reduced responses to sweet compounds following leptin administration in lean WT and *ob*/*ob* mice but not in *db*/*db* mice [64,65]. Moreover, the administration of a leptin antagonist conversely enhanced gustatory nerve responses to sweeteners in normally fed mice [66], further indicating the functional significance of leptin on sweet suppression.

**Figure 3:**
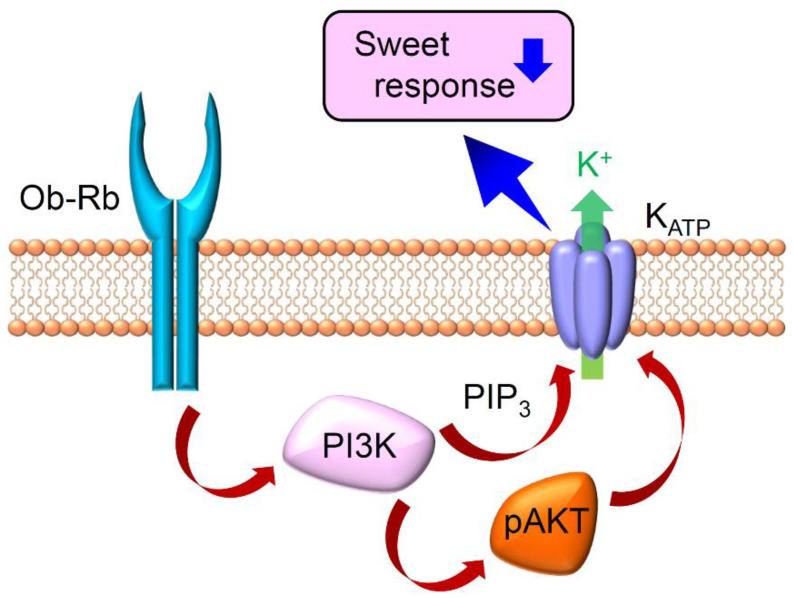
Schematic diagram showing molecular mechanisms for leptin signaling in sweet-sensitive taste cells. Leptin binding to the leptin receptor (Ob-Rb) induces the activation of phosphoinositide 3-kinase (PI3K), leading to the production of phosphatidylinositol (3,4,5)-trisphosphate (PIP_3_) and phosphorylation of AKT. These signaling components might activate K_ATP_ channels to induce cell hyperpolarization, leading to the suppression of sweet responses (indicated by blue down arrow).

As gustatory nerve responses to sweeteners were influenced by leptin administration, it was hypothesized that the target of leptin might be the peripheral taste organ, taste receptor cells. Indeed, in situ hybridization studies revealed the expression of functional leptin receptor *Ob-Rb* (*Leprb*) in the fungiform and circumvallate taste buds. [60,65]. Furthermore, the coexpression of *Leprb* and TAS1R3 in a subset of taste bud cells was demonstrated through a combination of in situ hybridization and immunohistochemistry [50]. Definitive evidence was provided by recording taste cell responses, where the bath application of leptin suppressed responses to sweeteners specifically in a subset of TAS1R3-positive taste cells, while responses to bitter compounds in gustducin-positive taste cells and sour compounds in GAD67-positive taste cells remained unaffected [50]. Similar to gustatory nerve recordings and behavioral assays, the effect of leptin was absent in the TAS1R3-positive taste cells of *db*/*db* mice, and the sweet suppressive effect of leptin was inhibited by a leptin antagonist. This sweet suppressive effect of leptin appears to be mediated by Ob-Rb expressed in a subset of TAS1R3-positive taste cells. Leptin’s mechanism of suppressing sweet taste responses in taste cells involves increasing the outward potassium current, as evidenced by patch clamp recordings from isolated taste cells [50]. This suggests that intracellular signaling activated by leptin binding to its receptor enhances the opening of K^+^ channels in sweet-sensitive taste cells. Notably, TAS1R3-positive cells possess components of the K_ATP_ channel, SUR1 and Kir6.1 [47], and leptin has been reported to activate K_ATP_ channels in various cell types [67,68,69]. Indeed, the K_ATP_ channel opener diazoxide mimicked the sweet suppressive effect of leptin, while the K_ATP_ channel blocker glibenclamide inhibited this effect, indicating the involvement of the K_ATP_ channel in leptin’s suppression of sweet responses in taste cells [50]. Recent research has elucidated the intracellular signaling pathway linking leptin receptor activation by leptin and K_ATP_ channel opening [70]. Phosphoinositide 3-kinase (PI3K) is one of the known signaling components activated after leptin receptor activation. Blocking PI3K diminished the sweet suppressive effect of leptin in TAS1R3-positive taste cells, and leptin stimulation led to the production of phosphatidylinositol (3,4,5)-trisphosphate (PIP_3_) and phosphorylation of AKT in a subset of TAS1R3-positive taste cells. A previous report demonstrated that PIP_3_ directly activates the K_ATP_ channel [71], and actin remodeling is involved in the PI3K-mediated activation of the K_ATP_ channel by leptin stimulation [72,73,74]. In summary, leptin suppresses sweet taste responses in TAS1R3-positive taste cells via the Ob-Rb–PI3K–K_ATP_ channel axis. The mechanisms for leptin signaling in sweet sensitive taste cells are summarized in Figure 3.

**Figure 4:**
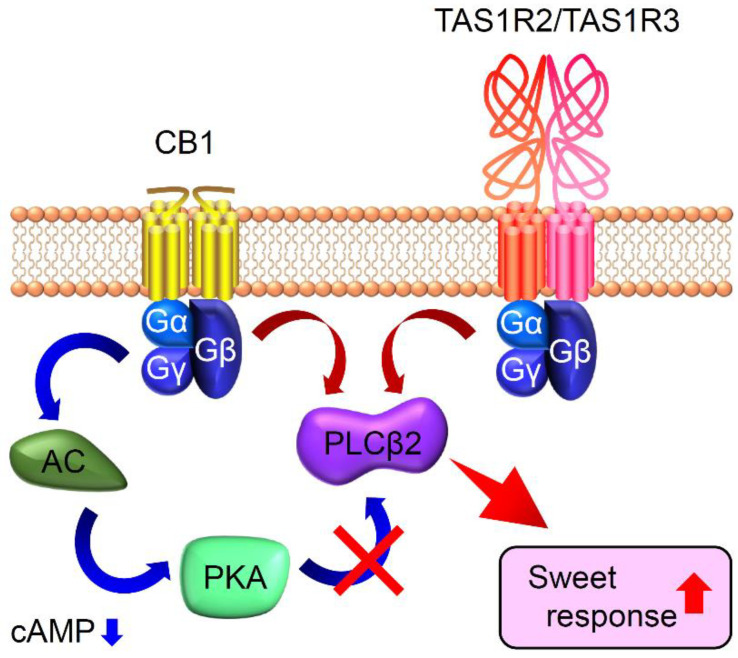
Schematic diagram showing possible molecular mechanisms for cannabinoid signaling in sweet-sensitive taste cells. Endocannabinoids binding to the cannabinoid receptor (CB_1_) activate phospholipase Cβ2 (PLCβ2) via the Gαq pathway. The synergistic or additive activation of PLCβ2 results in enhanced sweet responses in sweet-sensitive taste cells (indicated by red up arrow). Alternatively, the activation of CB_1_ suppresses adenylyl cyclase (AC) activity via the Gαi/o pathway, leading to a decrease in [cAMP]_i_ (indicated by blue down arrow). The reduction in cAMP levels decreases the activity of protein kinase A (PKA), leading to the disinhibition of PLC signaling (indicated by X) and enhanced sweet responses in sweet-sensitive taste cells.

On the other hand, certain bioactive substances enhanced sweet taste responses in mice. Endocannabinoids such as anandamide [N-arachidonoylethanolamine (AEA)] and 2-arachidonoyl glycerol (2-AG) are recognized as orexigenic regulators that stimulate appetite and food intake by acting on the hypothalamus and limbic forebrain [75,76,77]. In contrast to anorexgenic leptin, these orexigenic endocannabinoids enhanced sweet responses in mice [78]. The administration of AEA or 2-AG increased gustatory nerve responses to sweet tastants without affecting salty, sour, bitter, or umami taste responses. Corresponding to the enhancement in sweet responses in the gustatory nerve, lick responses to a sweet–bitter mixture were increased by the administration of AEA or 2-AG. This effect was absent in mice lacking CB_1_ receptors, suggesting that CB_1_ is indispensable for the sweet enhancement effect of endocannabinoids. In taste tissues, CB_1_ was coexpressed with TAS1R3, and the taste responses of TAS1R3-expressing cells were enhanced by AEA or 2-AG administration. Therefore, endocannabinoids modulate taste cell responses to sweeteners by activating CB_1_ on TAS1R3-expressing taste cells. The precise mechanism by which the CB_1_ signaling pathway enhances sweet taste responses remains unclear. CB_1_ is a G-protein-coupled receptor that primarily couples to Gi/o proteins, leading to the inhibition of adenylyl cyclase and a reduction in cAMP levels [79]. Regarding the cAMP level in taste cells, *Gnat3*-KO mice exhibited significantly elevated cAMP levels compared to WT mice, and elevated cAMP likely activated protein kinase A (PKA), which in turn phosphorylated and inhibited PLC signaling effectors, since treatment with a specific PKA inhibitor (H-89) restored responses to bitter stimuli in *Gnat3*-KO mice [80,81]. Although this mechanism applies to bitter taste, both bitter and sweet receptors share a similar signaling pathway. Therefore, reducing cAMP levels via the CB_1_ signaling pathway might decrease PKA activity, leading to the disinhibition of PLC signaling and enhanced sweet responses in TAS1R3-expressing cells (Figure 4). CB1 can also couple with other Gα proteins such as Gq. In this scenario, PLC could be activated via both TAS1R3-mediated and CB_1_-mediated pathways, further activating PLC and its downstream signaling. In summary, the enhancement in sweet responses by endocannabinoids likely involves TAS1R3-mediated sweet responses, rather than sugar responses mediated by glucose transporters in taste cells. Possible mechanisms for cannabinoid signaling in sweet sensitive taste cells are summarized in Figure 4.

Moreover, taste bud cells possess an additional mechanism that amplifies sweet taste responses, particularly to sugars. Adrenomedullin (ADM) is a peptide hormone with diverse functions including vasodilatory and hypotensive effects, the regulation of hormone secretion, the modulation of inflammatory responses, and influence on glucose metabolism [82]. The receptor components for ADM include calcitonin receptor-like receptor (CRLR) and receptor activity-modifying protein 2 (RAMP2) or RAMP3 [83]. The analysis of RNA-seq data from taste bud cells indicates the expression of these receptor components [84,85]. Indeed, CRLR and RAMP2 have been identified in TAS1R3-positive taste cells in mice [86]. The administration of ADM enhanced gustatory nerve responses to glucose and sucrose but not SC45647, an artificial sweetener. Furthermore, increased sugar responses were observed in *Tas1r3*-KO mice. Thus, it appears that the TAS1R3-dependent sweet signaling pathway is not affected by ADM. A potential mechanism contributing to the enhancement in sugar response involves glucose transporters expressed in taste cells, as the uptake of 2-NBDG was enhanced by ADM administration [86]. ADM has been reported to upregulate the expression of SGLT1 and the uptake of a non-metabolizable glucose derivative in rat intestines [87]. Consistent with this, treatment with ADM increased mRNA levels for SGLT1 in mouse fungiform and circumvallate taste buds [86]. Therefore, it is conceivable that ADM enhances the sugar responses of taste cells by increasing SGLT1 expression. However, further investigations are necessary to fully elucidate the mechanisms underlying the enhancement in sugar responses by ADM.

## 4. The Functions of Sweet Detection in the Oral Cavity

Carbohydrates, particularly sugars, are commonly utilized as a primary energy source in the body and are perceived as sweet in both rodents and humans. Consequently, the sweet taste is considered an inherently preferable signal for both rodents and humans. The preference for sweeteners has been evaluated through behavioral experiments in rodents. In a standard 48 h two-bottle preference test, animals are exposed to taste solutions for an extended period, potentially enabling them to recognize sugar solutions through sensory cues including taste, smell, texture, or visceral sensations, possibly forming associations between caloric content and these sensory inputs. In contrast, in short-term lick tests, animals are not exposed to taste solutions for as long, allowing for the analysis of simple taste reactions. Studies using both short-term lick tests and long-term (48 h or 24 h) two-bottle tests have shown that mice lacking *Tas1r2* or *Tas1r3* did not demonstrate a preference for artificial sweeteners such as acesulfame K, saccharin, sucralose, and SC45647 [17,18,88,89], indicating that TAS1R2 and TAS1R3 are crucial for detecting and preferring these sweet compounds. However, these KO mice maintained a preference for sugars such as sucrose and glucose, particularly at high concentrations in long-term two-bottle tests, although their preference was weaker compared to control WT mice [17,90,91]. In short-term lick tests, both *Tas1r2*-KO and *Tas1r3*-KO mice did not exhibit a significant preference for glucose and sucrose [88,89,90,91,92]. Similarly, *Trpm5*-KO mice showed no preference for sucrose in short-term lick tests but demonstrated a slight preference for sucrose compared to WT controls in long-term two-bottle tests [38,42,90]. In summary, the TAS1R2/TAS1R3-dependent signaling pathway appears essential for the innate preference for sweet substances (sugars, artificial sweeteners, etc.) in mice (Table 1). However, for the preference for sugars observed in long-term two-bottle tests, mechanisms independent of TAS1R2/TAS1R3, such as glucose transporters, may act as sugar sensors in the oral cavity. Additionally, other sensory signals, such as somatosensory or olfactory cues, may also contribute to the detection of sugars orally. Regarding olfactory signals, *Tas1r3*-KO mice, with or without bulbotomy (the surgical removal of the olfactory bulbs), displayed similar preferences for high concentrations of sucrose in long-term two-bottle tests [93], suggesting that olfactory cues may not be necessary for detecting sugars in the context of long-term preference tests.

The sensation of taste triggers various responses known as cephalic phase responses, including saliva and gastric acid secretion, stomach motility, thermogenesis, and hormonal secretions [130,131,132]. One notable response is cephalic phase insulin release (CPIR), where taste stimulation prompts an early release of insulin from pancreatic β-cells [133,134,135]. Particular stimuli eliciting CPIR in humans are sugars [136,137]. Furthermore, an artificial sweetener, saccharin, has been reported to induce CPIR in rats [133,138,139]. These results underscore the importance of sweet detection in initiating CPIR. Sweet detection involves at least two pathways: TAS1R2/TAS1R3 and glucose transporters. However, some studies suggest that non-nutritive sweeteners do not induce CPIR in humans and mice [140,141,142,143], indicating that TAS1R2/TAS1R3-mediated sweet detection may not be linked to CPIR initiation. Experiments with *Tas1r3*-KO mice revealed key insights. First, the gastric infusion of glucose into the gut failed to elicit CPIR in both WT and *Tas1r3*-KO mice [94,95], suggesting that gastric glucose alone does not suffice to trigger CPIR. Second, both WT and *Tas1r3*-KO mice displayed similar increases in plasma insulin five minutes after the oral ingestion of a glucose solution [94,95], indicating that oral glucose stimulation is crucial for CPIR induction and that TAS1R3-dependent receptors are not involved in CPIR initiation. Therefore, potential mechanisms for inducing CPIR involve oral glucose transporters. In support of this, the oral treatment of phlorizin and phloretin, which are pharmacological blockers of GLUTs and SGLTs, significantly inhibited a rapid increase in plasma insulin levels in WT mice after the oral ingestion of a glucose solution [95]. Conversely, the non-metabolizable glucose analog methyl-α-D-glucopyranoside (MDG) did not induce CPIR in mice [95,143], indicating that glucose metabolization within taste cells is likely necessary for CPIR induction. Similarly, *Abcc8* (SUR1)-KO mice did not exhibit CPIR, and pharmacological agents targeting K_ATP_ channels modulated the magnitude of CPIR in mice [143]. Given that the impairment of either glucose transporters or K_ATP_ channels attenuates CPIR, this suggests that neural signals stemming from the activation of the glucose transporter–K_ATP_ channel pathway in taste cells may be essential for inducing CPIR in mice (Table 1). However, the precise mechanism by which these glucose signals are relayed to the pancreas remains unclear. K_ATP_ channel subunits and glucose transporters are thought to be expressed in type II cells [47], which potentially use ATP as a neurotransmitter, with CALHM channels being crucial for ATP release [96,97]. Thus, sugar signals mediated by the glucose transporter–K_ATP_ channel pathway might be transmitted to gustatory nerve fibers via CALHM channels in taste cells and purinergic receptors on the nerve fibers. Interestingly, both *Calhm1*-KO and *P2x2*/*3*-double KO mice still exhibited CPIR in response to oral glucose stimulation, similar to their WT counterparts [143]. Therefore, glucose transporter-dependent sugar signals might be transmitted to gustatory nerve fibers via non-purinergic pathways from taste cells. Two potential candidates contributing to this signal transmission are peptidergic transmission, as glucagon-like peptide-1 is reported to be released from taste cells upon sweetener stimulation [144,145], and cholinergic transmission, given that taste cells release acetylcholine in response to sweet–bitter mixtures [146], and solitary chemosensory cells sharing similar properties with type II taste cells express choline acetyltransferase (Chat) [147]. Further investigations utilizing transgenic mice, such as *Glp1r*-KO mice and *Chat*-conditional KO mice, will enhance our understanding of the neural mechanisms underlying CPIR induction.

## 5. The Functions of Sweet Detection in the Intestine

Not only the oral cavity but also other organs, including the gastrointestinal tract, express the sweet taste receptor TAS1R2/TAS1R3 and glucose transporters. In the gastrointestinal tract, the expression of TAS1R2, TAS1R3, and gustducin was notably observed in enteroendocrine cells within the intestine [98]. These sweet receptors in the intestine likely play a role in regulating the expression of SGLT1. Studies on *Tas1r3*-KO and *Gnat3*-KO mice have shown that they did not exhibit a sugar-induced increase in SGLT1 expression. Conversely, the consumption of artificial sweeteners stimulated the expression of SGLT1 in the intestine of WT mice [98]. Furthermore, experiments with glucagon-like peptide-2 (GLP-2) receptor KO mice have revealed that the increase in intestinal SGLT1 expression due to sugar consumption was impaired in these mice [99]. This suggests that GLP-2 mediates the sugar-induced increases in SGLT1 expression in the intestine. Notably, TAS1R2 and TAS1R3 were coexpressed with GLP-2, and the small intestine of mice secreted GLP-2 in response to glucose and sucralose [99]. The GLP-2 receptor is primarily localized to enteric neurons [100], suggesting that sweetener-induced GLP-2 secretion could activate enteric neurons and subsequently influence SGLT1 expression in the intestine. Concerning intestinal sugar absorption, the trafficking of GLUT2 to the apical membrane of enterocytes is also upregulated by TAS1R2-mediated sugar detection and GLP-2 release within the intestine [101]. Consistent with these findings, the long-term consumption of sucralose in mice resulted in the upregulation of sweet receptors and glucose transporters’ expression [102,103]. These regulations of glucose transporters in the intestine via the activation of sweet receptors enhance glucose absorption from the luminal membrane, indicating that luminal sugar sensing by TAS1R2/TAS1R3 facilitates glucose absorption in the intestine (Table 1). However, unlike sweet perception in the oral cavity, changes in the expression levels of sweet receptors and glucose transporters may take some time, at least 4–5 days with high-carbohydrate diets or diets supplemented with sweeteners. Enteroendocrine cells expressing TAS1R2, TAS1R3, and gustducin also expressed GLP-1, and the increase in plasma GLP-1 levels following glucose administration was smaller in *Gnat3*-KO mice compared to WT controls [148]. Similarly, in humans, GLP-1 and TAS1R3 or gustducin were found to be colocalized in the intestine, and the inhibitor lactisole, which targets sweet taste receptors, suppressed the increase in plasma GLP-1 levels after glucose ingestion [149]. These findings suggest that the TAS1R-dependent sweet pathway contributes to GLP-1 release in the intestine. Indeed, experiments with the mouse endocrine cell line STC-1 demonstrated that GLP-1 was released in response to various sweeteners, and this response was inhibited by gurmarin, a rodent-specific inhibitor of sweet taste receptors [150]. The release of intestinal GLP-1 mediated by sweet taste receptors may be associated with metabolic diseases such as type II diabetes, as alterations in metabolic disorders can lead to changes in the gene expression of the sweet taste signaling pathway in the intestine, potentially contributing to impaired GLP-1 secretion [151].

In addition, sugar signals originating from the gastrointestinal tract contribute to behavioral preference and/or discrimination for sugars in animals. In long-term (24 h) tests, mice lacking TAS1R3 or TRPM5 still demonstrated strong preferences for sugar solutions [17,90,91]. These KO mice can also be conditioned to prefer flavored solutions when paired with intragastric infusions of sucrose or glucose [104,105]. Consequently, gastrointestinal sugar signals are necessary for learned avidity to sugars, and these signals do not rely on TAS1R-dependent receptor mechanisms. Furthermore, post-ingestive mechanisms are required for the learned preference for glucose over fructose through ingestive exposure to these sugars [106], alongside the necessity of olfactory cues [107,108]. Sugar signals from the intestine likely originate from neuropod cells, which are enteroendocrine cells forming glutamatergic excitatory synapses with vagal neurons [109]. This synaptic connection ensures rapid neuronal signaling within seconds. The stimulation of cholecystokinin (CCK)-positive duodenal neuropod cells with sugars or artificial sweeteners evoked responses in the cervical vagus nerve. These responses were inhibited by gurmarin (in response to sucralose) or phlorizin (in response to sucrose or α-methylglucopyranoside), indicating that both sweet taste receptors and sodium glucose transporters contribute to sugar or sweetener detection in CCK-positive neuropod cells. Moreover, sucrose intake in a 1 h two-bottle choice assay was suppressed by the optical silencing of CCK-positive neuropod cells, indicating that sugar preference depends on CCK-positive duodenal neuropod cells [110]. Intestinal stimulation with glucose, but not with acesulfame K and fructose, activated nodose ganglion neurons, and this activation was inhibited by phlorizin [111]. Collectively, these findings suggest that glucose transporters (SGLT1) in the intestine serve as receptors for sugars and contribute to sugar preference in mice (Table 1). These sugar signals are transmitted to the vagal nerve, then to nodose ganglion neurons, neurons in the nucleus of the solitary tract, and eventually to the parabrachial region and dopaminergic neurons in the substantia nigra [112], thereby establishing the neural reward pathway crucial for the development of sugar preference.

Glucose sensors in the intestine also contribute to the regulation of food intake (Table 1). In the hypothalamus, neurons expressing agouti-related protein (AgRP) become highly active during hunger, and the activation of these AgRP neurons promotes feeding behavior [113]. The activity of AgRP neurons was suppressed by glucose infusion into the duodenum, and this suppression was blocked by the SGLT1/3 inhibitor phlorizin but not by the GLUT2 inhibitor phloretin. Additionally, the activity was reduced by splanchnic lesion but not by a complete subdiaphragmatic vagotomy [114]. This indicates that intestinal sugars activate the splanchnic nerve via the SGLT1/3-dependent sugar detection system, and sugar information is transmitted to hypothalamic AgRP neurons through the splanchnic nerve. Interestingly, the optogenetic activation of AgRP neurons innervating the lateral hypothalamus increased preference for sucrose [115]. Thus, complex neural circuits are formed among the oral cavity, gut, and brain to optimize caloric intake and maintain energy homeostasis in the body.

## 6. The Functions of Sweet Detection in the Pancreas

Insulin, an anabolic hormone, is secreted from β-cells in the pancreatic islets in response to glucose. This process begins with glucose uptake into the β-cell via GLUTs, where glucose is metabolized to produce ATP, resulting in an increase in intracellular ATP concentration ([ATP]_i_). Subsequently, the closure of K_ATP_ channels occurs, leading to the depolarization of the β-cell. Adequate depolarization triggers the generation of action potentials, which in turn opens voltage-dependent Ca^2+^ channels, facilitating the influx of Ca^2+^ and inducing the exocytosis of insulin-containing vesicles [120]. Although the GLUT-K_ATP_ channel pathway is indispensable for insulin release, TAS1R-dependent sweet detection plays a part in regulating insulin secretion (Table 1). TAS1R3 was coexpressed with insulin in the islets of mice, and other TAS1R-dependent components such as TAS1R2 and gustducin were also detected at the mRNA level within the islets [116]. The stimulation of a mouse pancreatic β-cell line (MIN6 cells), which also expresses TAS1R3, with sucralose enhanced the glucose-induced increase in [ATP]_i_. The knockdown of *Tas1r3* abolished this potentiation [117], suggesting that TAS1R3-mediated sweet detection could enhance K_ATP_ channel closure in β-cells, thereby increasing insulin secretion. Indeed, sucralose can induce bioelectrical activity in β-cells at a low (3 mM) concentration of glucose, although sucralose alone (without glucose) was insufficient to elicit such activity [118]. Thus, TAS1R-dependent mechanisms likely collaborate with glucose transporters to augment insulin secretion from pancreatic β-cells. However, the molecular mechanisms underlying the interaction between the TAS1R3-dependent pathway and glucose transporters remain unclear, necessitating a detailed analysis of the intracellular signaling pathways following TAS1R3-dependent receptor activation. Furthermore, another study observed the potentiation of calcium response and insulin release in β-cells upon fructose stimulation [119]. This effect was absent in β-cells lacking *Tas1r2*. The downstream components implicated in this response are PLC and TRPM5, as the PLC blocker U73122 abolished the fructose-induced calcium response in β-cells, and β-cells lacking *Trpm5* showed no potentiation of calcium responses by fructose [119]. These findings suggest that multiple mechanisms contribute to the TAS1R-dependent regulation of insulin release from β-cells.

## 7. The Functions of Sweet Detection in the Brain

The brain, being the most energy-intensive organ in the human body, plays a pivotal role in regulating energy intake, particularly through the hypothalamus. This brain region is crucial for controlling feeding and energy expenditure. Within the hypothalamus, there are two types of glucose-sensitive neurons: glucose-excited and glucose-inhibited neurons [124]. The primary glucose detection system in these neurons relies on GLUTs. In glucose-excited neurons, glucose enters through GLUT2 and is metabolized by glucokinase to produce ATP. Increased ATP levels lead to the closure of K_ATP_ channels, causing cell depolarization. Conversely, in glucose-inhibited neurons, reduced extracellular glucose decreases glucose uptake via GLUT2, lowering ATP levels required for the Na^+^/K^+^ ATPase pump’s activity. This reduced pump activity causes intracellular Na^+^ accumulation, leading to cell depolarization. Additionally, in glucose-inhibited neurons, AMP-activated protein kinase (AMPK), neuronal nitric oxide synthase (nNOS), soluble guanylate cyclase (sGC), and Cl^−^ channels contribute to cell depolarization due to decreased ATP production from reduced glucose uptake via GLUT2 [125]. Besides the glucose transporter-dependent system, TAS1R3-dependent receptors also play a role in regulating neural activity in these hypothalamic neurons (Table 1). Immunohistochemical studies have shown the expression of TAS1R2 and TAS1R3 in the arcuate nucleus (ARC) of the hypothalamus [121]. Indeed, certain ARC neurons showed responses to the artificial sweetener sucralose, and the inhibition of sweet receptors by gurmarin suppressed high-glucose responses in glucose-excited neurons [122]. The sucralose-activated neurons were primarily non-pro-opiomelanocortin (POMC) neurons, and their activation by sucralose injection into the brain’s ventricles reduced food intake [122]. Thus, TAS1R-dependent sweet detection in hypothalamic neurons contributes to the regulation of food intake. Hypothalamic neurons, hypothalamic tanycytes, and glial-like glucose-sensitive cells also responded to non-nutritive sweeteners like sucralose and acesulfame K. The proportion of glucose-sensitive tanycytes was notably reduced in *Tas1r2*-KO mice compared to WT mice, indicating the likely involvement of TAS1R-dependent receptors in tanycytes [152]. However, the physiological significance of sweet responses mediated by sweet receptors in tanycytes remains unclear.

TAS1Rs are also expressed in other brain regions. For example, neurons in the granule cell and pyramidal cell layers of the hippocampus exhibit a clear expression of mRNAs and proteins for gustducin, TAS1R2, and TAS1R3 [121,153]. Studies on *Tas1r3*-KO mice demonstrated that the absence of TAS1R3 in hippocampal neurons leads to increased neuritic density, reduced spine density, and longer dendrites, suggesting neurological dysfunction and/or synaptic failure in these neurons. Correspondingly, *Tas1r3*-KO mice exhibited altered learning and memory functions compared to WT mice [123]. Thus, TAS1R-dependent signals may be required for learning and memory functions in the brain (Table 1). Additionally, astrocytes positive for glial fibrillary acidic protein (GFAP) in the hippocampus of rats began to express TAS1R2, TAS1R3, and gustducin following ischemic injury [153]. Although ischemic injury triggers the expression of these sweet receptor/transduction components in reactive astrocytes, their specific functions in astrocytes remain unknown. Other brain regions expressing TAS1R2 are revealed by using *Tas1r2*-Cre x reporter mice [154]. These regions include the circumventricular organs, vascular structures in the cortex, thalamus, and striatum. Further investigations are needed to understand the functions of these TAS1R-dependent receptor systems expressed in the brain.

## 8. Functions of Sweet Detection in Other Organs

Chemosensory cells are present in the respiratory epithelium of both the upper and lower airways. These cells, known as solitary chemosensory cells (SCCs), express taste receptors and associated transduction components, including TAS2Rs, TAS1R3, gustducin, and TRPM5 [155,156]. SCCs respond to bitter compounds and acyl-homoserine lactones (AHLs) produced by Gram-negative bacteria. When stimulated by these bitter compounds or AHLs, SCCs significantly decreased respiration via the activation of the trigeminal nerve [155,157,158]. Therefore, the bitter taste signaling in airway SCCs plays a role in suppressing respiration. Additionally, bitter signaling in SCCs is involved in innate immune defense. The bitter compound denatonium activates a subset of airway SCCs, inducing calcium responses that propagate through gap junctions to surrounding respiratory epithelial cells. This process leads to the release of antimicrobial peptides such as beta-defensin 1 and 2 [126]. Interestingly, both bitter and sweet taste receptors are often coexpressed in the same SCCs [126,156,159]. The activation of the sweet receptor (TAS1R2/TAS1R3) suppresses the bitter-induced secretion of antimicrobial peptides [126]. This suppression by sweeteners (glucose, sucrose, and sucralose) was reduced by lactisole but not by phloretin and phlorizin, indicating the involvement of TAS1R-dependent receptors in the suppression of antimicrobial peptide secretion by bitter compounds. It is hypothesized that sweet receptors are tonically activated by low levels of glucose in the airway surface liquid under normal conditions, thus suppressing antimicrobial peptide secretion. During bacterial infection, the glucose level in the airway surface liquid decreases as bacteria consume the sugar. This reduction triggers the disinhibition of antimicrobial peptide secretion, helping to prevent bacterial proliferation (Table 1). However, the intracellular mechanisms following the activation of sweet taste receptors in SCCs remain unknown. Additionally, it is uncertain whether sweet receptors can detect 0.5 mM glucose. There might be additional mechanisms involved in detecting low glucose concentrations.

The TAS1R2/TAS1R3 receptor system may also contribute to postnatal bone remodeling (Table 1) [127]. During their investigation of the adipose tissue phenotypes of *Tas1r2*- and *Tas1r3*-KO mice, Simon et al. discovered that these KO mice exhibited increased cortical bone mass and trabecular remodeling after consuming a Western diet for 14–24 weeks [128]. Similarly, 20-week-old *Tas1r3*-KO mice on a normal diet showed a modest increase in the average thickness of the cortical bone [129]. Bone homeostasis is maintained by a balance between bone resorption by osteoclasts and bone formation by osteoblasts. Therefore, the increased cortical bone mass in *Tas1r3*-KO mice suggests enhanced bone formation and/or reduced bone resorption. RT-PCR experiments confirmed the expression of both *Tas1r2* and *Tas1r3* in differentiated primary murine osteoclasts. In *Tas1r3*-KO mice, the serum levels of the bone resorption marker collagen type I C-telopeptide (CTx) were lower compared to WT mice. In WT mice, increased *Tas1r3* expression strongly correlated with elevated levels of the mature osteoclast marker Cathepsin K. However, the serum levels of the bone formation marker procollagen type 1 N-terminal propeptide (PINP) in *Tas1r3*-KO mice did not differ from those in WT mice [129]. Taken together, the reduction in bone resorption observed in *Tas1r3*-KO mice may be due to impaired osteoclast differentiation caused by the absence of TAS1R3. Nevertheless, the precise mechanisms through which TAS1R3-dependent signaling influences osteoclast function remain unknown.

## 9. Conclusions and Future Directions

Sweet receptor systems are present throughout the body. Their primary role is believed to be the regulation of energy homeostasis, as they are involved in glucose absorption, insulin release, sugar preference, and food intake. Therefore, sweet receptor systems could be clinical targets for treating metabolic disorders such as obesity and diabetes. For instance, suppressing TAS1Rs in the intestine could reduce intestinal SGLT1 expression, thereby decreasing sugar absorption and potentially combating obesity. Intestinal glucose transporters could be targeted to suppress sugar signals from the intestine, which may help reduce the preference for sugars. In the future, the development of treatments for metabolic disorders mediated by sweet receptors is anticipated. While the molecular mechanisms of sweet detection in taste cells are well understood, the intracellular signaling pathways following the activation of sweet receptors—both TAS1R-dependent and -independent—remain unclear, particularly in extraoral organs. Furthermore, the functions of sweet receptors in some organs have not yet been elucidated. Given the growing evidence of extraoral sweet receptors, these sweet sensing systems may have additional unknown functions in tissues that have not yet been studied. Understanding sweet sensing systems in the body could provide valuable clinical insights for the treatment of various diseases.

## Figures and Tables

**Table 1:** Functions of sweet reception in oral and extra-oral organs.

Organ	Receptor	Function	Reference
Oral cavity	TAS1Rs	an inherently preferable signal	[17,18,88,89,90,91,92]
	GLUTs/SGLTs	cephalic phase insulin release	[94,95,96,97]
Intestine	TAS1Rs	enhancement of glucose absorption	[98,99,100,101,102,103]
	SGLTs	preference for sugars	[104,105,106,107,108,109,110,111,112]
	SGLTs	regulation of food intake	[113,114,115]
Pancreas	TAS1Rs	regulation of insulin secretion	[116,117,118,119]
	GLUTs	insulin secretion	[120]
Brain	TAS1Rs	regulation of neural activity (food intake)	[121,122]
	TAS1Rs	learning and memory functions	[123]
	GLUTs	regulation of neural activity (food intake)	[124,125]
Respiratory epithelium	TAS1Rs	prevention of bacterial proliferation	[126]
Bone	TAS1Rs	bone remodeling	[127,128,129]

## Abbreviations

2-AG	2-arachidonoyl glycerol
2-NBDG	2-[N-(7-Nitrobenz-2-oxa-1,3-diazol-4-yl)amino]-2-deoxy-D-glucose
AC	adenylyl cyclase
ADM	adrenomedullin
AEA	N-arachidonoylethanolamine
AgRP	agouti-related protein
AHL	acyl-homoserine lactone
AMPK	AMP-activated protein kinase
AP	action potential
ARC	arcuate nucleus
ATD	amino-terminal domain
CCK	cholecystokinin
Chat	choline acetyltransferase
CPIR	cephalic phase insulin release
CRD	cysteine-rich linker domain
CTx	collagen type I C-telopeptide
GFAP	glial fibrillary acidic protein
GLP-1(or 2)	glucagon-like peptide-1 (or 2)
GLUT	glucose transporter
IP_3_	inositol-1,4,5-triophosphate
IP_3_R3	inositol-1,4,5-triophosphate receptor type 3
Kir6.1	inwardly rectifying potassium channel 6.1
KO	knockout
MDG	methyl-α-D-glucopyranoside
nNOS	neuronal nitric oxide synthase
PI3K	phosphoinositide 3-kinase
PINP	procollagen type 1 N-terminal propeptide
PIP_3_	phosphatidylinositol (3,4,5)-trisphosphate
PKA	protein kinase A
PLCβ2	phospholipase Cβ2
POMC	pro-opiomelanocortin
SCC	solitary chemosensory cell
sGC	soluble guanylate cyclase
SGLT	sodium–glucose cotransporter
SUR1	sulfonylurea receptor 1
TAS1R2(or 3)	taste receptor family 1 members 2 (or 3)
TMD	transmembrane domain
TRPM5	transient receptor potential channel M5
VFTM	Venus flytrap module
VGSC	voltage-gated sodium channel
WT	wild-type
